# *De novo* assembly of the dual transcriptomes of a polymorphic raptor species and its malarial parasite

**DOI:** 10.1186/s12864-015-2254-1

**Published:** 2015-12-09

**Authors:** Martina Pauli, Nayden Chakarov, Oliver Rupp, Jörn Kalinowski, Alexander Goesmann, Michael D. Sorenson, Oliver Krüger, Joseph Ivan Hoffman

**Affiliations:** Department of Animal Behaviour, Bielefeld University, 33501 Bielefeld, Germany; Bioinformatics and Systems Biology, Justus-Liebig-University, 35390 Giessen, Germany; Center for Biotechnology, Bielefeld University, 33501 Bielefeld, Germany; Department of Biology, Boston University, Boston, MA 02215 USA; Present address: Molecular Ecology and Evolution Lab, Lund University, 223 62 Lund, Sweden

**Keywords:** Transcriptome, Non-model organism, Common buzzard *Buteo buteo*, Plasmodium, Melanin pathway, Molecular markers

## Abstract

**Background:**

Studies of non-model species are important for understanding the molecular processes underpinning phenotypic variation under natural ecological conditions. The common buzzard (*Buteo buteo*; Aves: Accipitriformes) is a widespread and common Eurasian raptor with three distinct plumage morphs that differ in several fitness-related traits, including parasite infestation. To provide a genomic resource for plumage polymorphic birds in general and to search for candidate genes relating to fitness, we generated a transcriptome from a single dead buzzard specimen plus easily accessible, minimally invasive samples from live chicks.

**Results:**

We not only *de novo* assembled a near-complete buzzard transcriptome, but also obtained a significant fraction of the transcriptome of its malaria-like parasite, *Leucocytozoon buteonis*. By identifying melanogenesis-related transcripts that are differentially expressed in light ventral and dark dorsal feathers, but which are also expressed in other regions of the body, we also identified a suite of candidate genes that could be associated with fitness differences among the morphs. These include several immune-related genes, providing a plausible link between melanisation and parasite load. qPCR analysis of a subset of these genes revealed significant differences between ventral and dorsal feathers and an additional effect of morph.

**Conclusion:**

This new resource provides preliminary insights into genes that could be involved in fitness differences between the buzzard colour morphs, and should facilitate future studies of raptors and their malaria-like parasites.

**Electronic supplementary material:**

The online version of this article (doi:10.1186/s12864-015-2254-1) contains supplementary material, which is available to authorized users.

## Background

High-throughput DNA sequencing is transforming our understanding of the molecular mechanisms underlying physiological, morphological and even behavioural traits [1, 2]. In particular, although laboratory organisms make excellent models for understanding links between genotype and phenotype, new sequencing approaches have opened the way to studying natural populations [3–6]. This has brought more varied and realistic biological systems into reach and offers the opportunity to explore genotype-environment interactions.

High sequencing costs and a paucity of reference genomes initially restricted large-scale sequencing and marker development to a handful of model systems [7, 8]. However, these limitations have largely disappeared thanks to technological advances that allow increasing amounts of data to be generated at tumbling costs [1, 2]. Moreover, by directing sequencing effort towards the transcriptome, which is usually an order of magnitude smaller than the full genome, costs and downstream bioinformatic analysis can be further reduced.

A large avian order that differs considerably from common model species and for which few genomic resources are available is the Accipitriformes (comprising buzzards, hawks, eagles, harriers, kites, vultures and osprey). This group is globally distributed across all of the continents except for Antarctica [9]. As top predators and scavengers, these species are of great ecological importance, and many are of conservation concern [10]. Raptors are also interesting from the perspective of plumage colour polymorphism, which is found in many member genera including most *Buteo* hawk and buzzard species [9]. This often takes the form of discrete colour morphs, which not only differ in the extent of plumage melanisation, but often also in important fitness-related traits [11–15].

Melanin-based colouration is widespread throughout the animal kingdom, with the pigments involved ranging from yellow, through orange and red to brown and black [16]. These pigments are produced in specialised organelles, the melanosomes [17]. Polymorphisms within a transmembrane receptor of these organelles, the melanocortin 1 receptor (MC1R), have been linked to variation in plumage or pelage colouration in several species of birds and mammals [reviewed by 18]. However, the molecular mechanisms linking colouration to fitness remain unclear. This is partly due to the fact that mutations at this locus are believed to have minimal pleiotropic effects [18, 19].

**Fig. 1:**
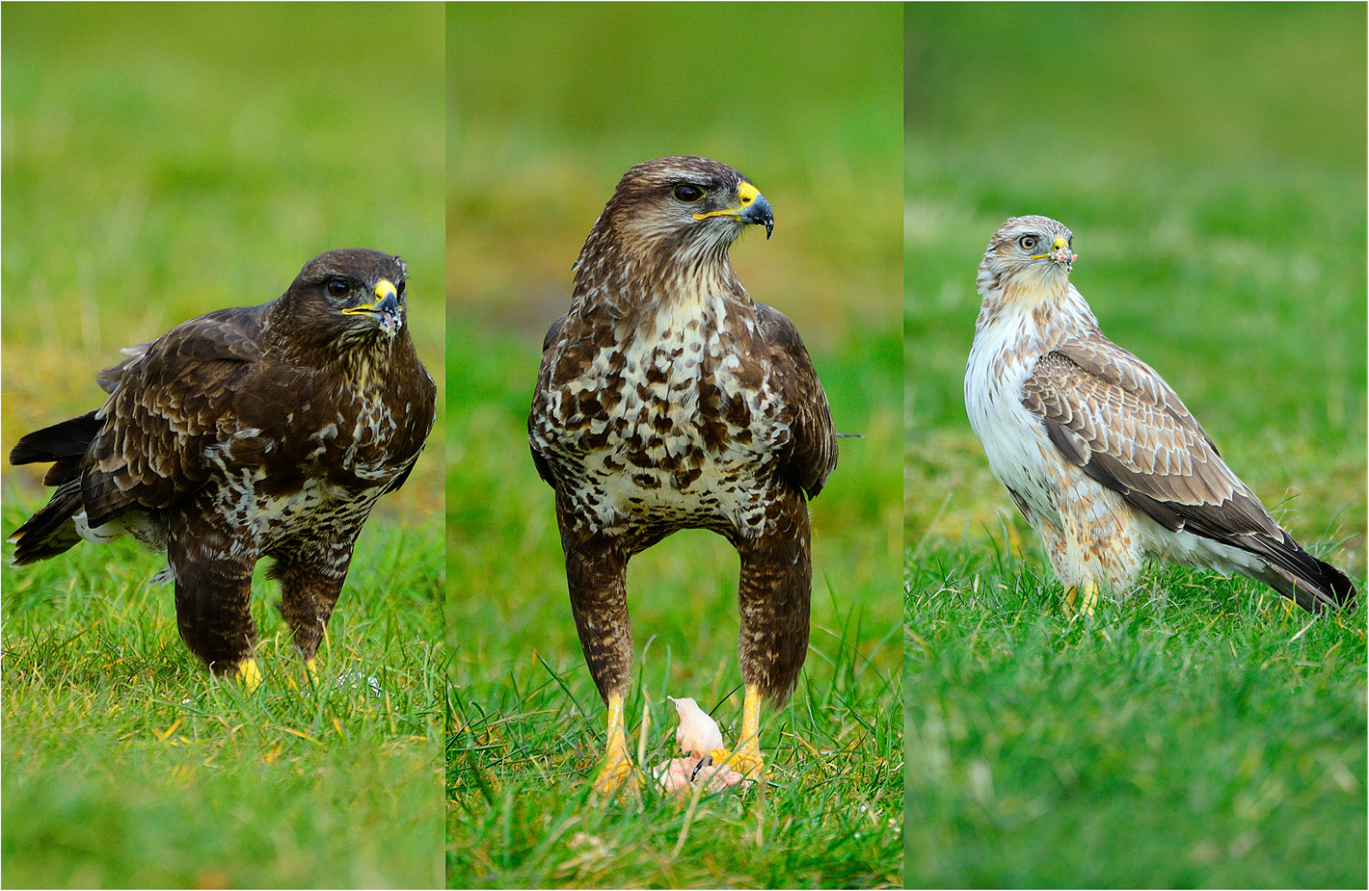
The three morphs of the common buzzard. The dark morph (*left*) has dark head, heavily speckled or dark breast and underwing coverts. Intermediate buzzards (*middle*) have dark head, intermediately speckled breast and underwing coverts. Light buzzards (*right*) have little or no melanisation of breast and underwing coverts, in extreme cases with light head and upperwing coverts

An ideal system in which to investigate the link between variation at the MC1R and fitness is provided by a long-term study of common buzzards (*Buteo buteo*). This species comprises three colour morphs termed ‘light’, ‘intermediate’ and ‘dark’ (shown in Fig. 1), which differ in lifetime reproductive success [12], parasite clone composition and infestation levels [11, 20] and aggressiveness [21]. Colour heritability follows Mendelian expectations for a single locus with two alleles [19], with the presumed heterozygote morph (intermediate) achieving the greatest lifetime reproductive success [16]. However, a recent study found that the plumage morphs do not differ in genome-wide heterozygosity, estimated from 18 microsatellites [22]. Likewise, multilocus heterozygosity does not appear to be significantly associated with body condition and only explains variation in parasite infection via complex interactions with age and prey abundance [22].

One intriguing possibility is that fitness differences among the morphs may relate to the differential effects of parasites on the three colour morphs. The dark morph has a tendency to be more heavily infested with the blood sucking fly *Carnus haemapterus* while the lighter morphs tend to carry higher loads of the malaria-like blood parasite *Leucocytozoon buteonis* (formerly known as *L. toddi*) [11]. *Leucocytozoon* in particular can dramatically reduce host fitness [23–25] by causing anaemia and organ damage [26]. *Leucocytozoon* is closely related to malaria-causing *Plasmodium* [27] and has a similar life-history involving stages in the liver and blood cells of the vertebrate host [26].

Here, we construct a *de novo* transcriptome for the common buzzard, thereby generating the first genomic resource for a plumage-polymorphic member of the Accipitriformes (see [28] for the genomes of two species of Falconiformes, a divergent and independently evolved group of predatory birds [29, 30]). In parallel, we also partially sequenced and *de novo* assembled the parasitic *L. buteonis* transcriptome, which is again a first for this genus. To achieve this, we sampled RNA from growing feathers, circulating blood and several different organs, including the liver, which is the primary host organ of early *Leucocytozoon* infection stages. As a first application of this new resource, we also analysed tissue-specific patterns of transcript presence and absence in order to identify transcripts involved in melanogenesis that might also affect biological processes beyond plumage colouration. Using this approach, we identified several candidate genes that could be tested for a role in morph-specific fitness differences.

## Methods

### Sampling

All samples were collected from nestling buzzards (*Buteo buteo*) in a study population in western Germany during the breeding season of 2012. Trees containing nests were climbed and the chicks were lowered to the ground to collect feather and blood samples. Samples were obtained from chicks selected to equally represent all three colour morphs. To allow quantification of differences in feather gene expression relating to colour, we sampled RNA from both dorsal (dark) and ventral (light) contour feathers. Feather samples were transferred to RNAlater immediately and stored at room temperature for up to 12 h before being frozen at –80 °C. For comparison, approximately 300μl of blood was taken from the brachial vein, transferred to RNAlater immediately and stored at room temperature for up to 12 h before again being frozen at –80 °C.

We also collected samples from the lung, liver, skin, heart muscle, breast muscle and brain of a single chick of the light morph that had fallen from its nest before we climbed the tree and which died of its injuries. The chick was frozen whole at –80°C within a few minutes of death and transferred to the laboratory, where each organ was dissected while the rest of the body was kept frozen. All tissue samples were taken within 5 min of the respective organ having been thawed, and were then transferred immediately to RNAlater where they were stored at –80 °C.

### RNA isolation, cDNA generation and sequencing

RNA was extracted by a guanidinium thiocyanate-phenol-chloroform method using TRIzol® (Life Technologies). Samples from organs and feathers were dry-blotted and immediately ground up in liquid nitrogen. Extraction then followed the standard TRIzol® protocol. Blood samples were first centrifuged at 12,000 g for 15 min and the supernatant RNAlater discarded. The remaining sample was then lysed and homogenized in 1ml of TRIzol® by repeated pipetting before continuing with the standard protocol. The resulting RNA extracts were then used to create four pools: *blood*, *ventral feathers*, *dorsal feathers* and *organs*. We did not prepare separate sub-libraries for the three colour morphs as this would have required a total of twelve normalised libraries, which was beyond the scope of our budget. The *blood* pool contained equal amounts of RNA from each of 30 different individuals, 10 of each morph. *Dorsal* and *ventral feathers* each contained equal amounts of RNA from 15 individuals, five of each morph. The *organs* pool contained equal amounts of RNA from each tissue type collected from the single dead chick. The RNA concentrations of each sample were measured on a Qubit (Life Technologies). Final RNA concentrations of the four pools were measured on a Bioanalyzer (Agilent). cDNA generation, library barcoding (one barcode each for *organs*, *blood*, *dorsal* and *ventral feathers*) and normalisation of the four pools was performed by vertis Biotechnologie (Freising, Germany). The normalisation was based on a kinetic denaturation-reassociation technique described at http://www.vertis-biotech.com/index.php?ip=132. A single MiSeq run was then conducted at the Center for Biotechnology, Bielefeld University.

### Quality filtering

The quality of the reads was checked using the FastQC toolkit (http://www.bioinformatics.babraham.ac.uk/projects/fastqc/). The FastQC analysis identified biased nucleotide content in the first 15 and last 10 bases. We therefore trimmed the first 15 and the last 10 bases of every read together with any other low-quality regions (Phred < 15).

### Sequence assembly

To maximise data quality and the inclusion of sequences representing genuine transcripts, we used a three-step assembly strategy. First, we assembled the quality-filtered reads *de novo* using Trinity version r2013-02-25 [31] and obtained additional assemblies based on different *k*-mer sizes using Velvet version 1.1.07 and Oasis version 0.2.08 [32, 33]. These programs were run with all odd *k*-mer values between 21 and 121 and default parameters, creating 51 additional *de novo* assemblies. In the second step, the contigs from all 52 assemblies combined were screened for likely protein-coding regions (CDS). All possible open reading frames (ORFs) were extracted using the TransDecoder tool included in the Trinity package. The translated protein sequences of all ORFs were mapped to the zebra finch reference protein set using blat [34]. The results were screened for hits that covered both the ORF and the reference protein by 100 % without any gaps. If more than one hit was found for a given reference protein, one was randomly chosen. These ORFs were then used as a training set to create the hexamer score used by TransDecoder. Additionally, all ORFs were searched against the Pfam-A database using the hmmscan tool [35]. All sequences lacking a likely CDS were discarded. In the final step, all predicted CDS sequences were translated to protein sequences and clustered using cd-hit version 4.6 [36] with 95 % global sequence identity (parameter -G 1 -c 0.95), keeping the longest sequence of each cluster in the final data set.

Screening the contigs for CDS and selecting representative sequences from each cluster should improve overall data quality, but it might also lead to the loss of some transcripts. To estimate the extent of this possible loss, we compared both the reduced and complete set of contigs with 15,431 zebra finch Ref Seq proteins (http://www.ncbi.nlm.nih.gov/bioproject/PRJNA32405) using BLAT [34]. We then counted how many zebra finch proteins aligned to our contigs with 80% coverage and a maximum of 5 % gaps.

### BLAST mapping, sequence annotation and comparative genomics

The final data set was uploaded to the SAMS system [37] and an automatic functional annotation was performed using a best blast hit strategy against various databases including SwissProt [38], KEGG [39] and KOG [40]. Additionally, the translated protein sequences were blasted against the chicken and zebra finch RefSeq proteins with known genomic locations. Since buzzards are known to have a high prevalence and often high infection intensities of the malaria-like parasite *Leucocytozoon buteonis* [11, 41] translated protein sequences were also blasted against the genomes of three human malarial species, *P. falciparum*, *P. vivax* and *P. knowlesi*. The blast results were filtered with an e-value cutoff of 10^-5^. Each transcript was assigned to a chicken and zebra finch chromosome on the basis of the top blast hit.

### Mining for molecular markers

We searched for microsatellites containing six or more repeat units using sputnik with the default parameters. For SNP detection, reads were mapped to the transcripts separately for each pool using the “alignReads.pl” script from the Trinity distribution within Bowtie2 [42]. SNPs were called using samtools [43]. Any SNPs with Phred quality scores below 15 were discarded.

### Assigning transcripts to the four pools

As the libraries had been normalised, it was not possible to analyse quantitative differences in transcript abundance among the different pools. We therefore focused on tissue-specific patterns of transcript presence and absence. To assign a transcript as ‘present’ in one of the four pools, we counted the percentage of bases covered by reads from this sample, using samtools depth [43] on the “alignReads.pl” mappings. If at least 80 % of bases were covered by at least one read, the transcript was considered to be present in the sample.

### Transcripts found in different tissues

We compared the overall presence / absence patterns of transcripts corresponding to known genes in the zebra finch among the four pools by annotating transcripts with respect to the RefSeq database at NCBI. Given that some of the pools were generated from different sample sizes of individuals, we expect there to be some level of error associated with certain comparisons. As the buzzard colour morphs differ in fitness, we were particularly interested in differences involving transcripts related to melanogenesis. We therefore extracted 86 proteins from the zebra finch melanin pathway from the KEGG database (http://www.kegg.jp/kegg/pathway.html) and used BLASTp to match our transcripts to these genes. Based on the premise that the colour morphs are defined by the extent to which they grow dark and light feathers, we then compared transcripts associated with melanogenesis between *dorsal* (dark) and *ventral* (light) feathers, as a proxy for morph, in order to identify putative candidate genes that might be involved in morph-specific fitness differences. As fitness-relevant genes are expected to be expressed not only in feathers, we then cross-referenced these transcripts with the *organs* pool to derive two subsets of transcripts from the melanogenesis pathway, one occurring in only *ventral* feathers and *organs*, and the other occurring in only *dorsal* feathers and *organs*. These were then queried for GO slim categories and involvement in biological pathways, according to KEGG.

### qPCR analysis

To verify patterns identified from the transcriptomic data, we performed qPCR analyses for seven of the differentially expressed genes in 20 samples (see Additional file 1: Table S1 for details). The genes that we tested were *ADORA2A*, *FZD3*, *FZD4*, *LIMK1*, *RAC1*, *STK4* and *WNT4*. Additionally, as a control that is unrelated to melanogenesis, we quantified the expression of the gene *Clock* [20]. cDNA from single feathers was synthesized with peqGold cDNA-Synthesis Kit H Plus. The qPCR was performed in a CFX Connect Real Time PCR Detection System (BioRad) using 96-well plates. The 10μl reaction volume contained 2 μl EvaGreen (qPCR-MixII, my-Budget), 1.25 μM of each primer and 0.1 μl of feather cDNA. Each qPCR reaction was performed in duplicate. Cq scores for each reaction were divided by the mean Cq score of the respective sample for *Clock*. Relative expression of separate genes relative to *Clock* was analysed with mixed-effects models incorporating sample, plate and gene identity as random factors and morph and body region of the feather as fixed factors. Factor significance was estimated by comparing models with likelihood ratio tests after exclusion of a fixed factor.

## Results

### Total RNA yields

We characterised the common buzzard transcriptome by creating four pools of RNA samples: *blood*, *organs*, *ventral feathers* and *dorsal feathers*. Organ samples (lung, liver, skin, heart muscle, breast muscle and brain) were obtained from a single dead chick to achieve as complete a transcriptome as possible without sacrificing individuals. Average RNA yields were highest from the *organs* (1089 ng/μl) followed by *ventral* and *dorsal feathers* (802 ng/μl and 761 ng/μl, respectively) and lowest from *blood* (286 ng/μl). Samples were pooled in roughly equimolar amounts into tissue-specific libraries that were then normalised prior to sequencing.

### Sequence data and transcriptome assembly

**Fig. 2:**
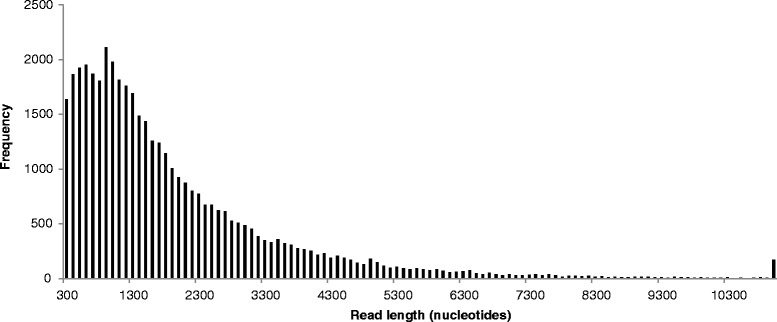
Transcript length distribution, pooled from all four libraries (o*rgans*, *dorsal feathers*, v*entral feathers* and *blood*)

A single MiSeq sequencing run generated 24,478,009 reads with an average length of 217 bp (median: 229 bp, std. dev. = 31.85). After quality filtering the reads, we applied a three-step assembly strategy. First we performed 52 individual *de novo* assemblies, including one Trinity [31] and 51 Velvet/Oases [32, 33] assemblies with the *k*-mer parameter varying from 21 to 121. Each of these assemblies comprised in the order of 95,000 to 567,000 contigs, and a total of 2,055,004 contigs were assembled. To filter out contigs that were either misassembled, contained only UTR sequence or were assembled from premature mRNA, we then extracted likely coding regions (CDS) using the “TransDecoder” tool from the Trinity package (see Methods for details). Discarding all transcripts without a likely CDS, the total number of contigs was reduced to 800,402. Finally, we filtered out redundant sequences by clustering the translated protein sequences with 95 % global sequence identity, which resulted in 42,875 clusters. Retaining the longest sequence per cluster reduced the final data set to 42,875 sequences (Fig. 2).

### Estimating data loss

Although filtering and clustering should improve overall data quality by enriching the pool of sequences for genuine transcripts, it is also possible that some transcripts might be inadvertently lost. To estimate the extent of this possible loss, we aligned 15,431 reference zebra finch proteins to the initially assembled sequences and to the final dataset. We then counted the numbers of reference proteins with a best bidirectional hit covering at least 80 % of the reference protein with at most 5 % gaps. In the initially assembled sequences, 6,231 reference proteins could be found, whereas 5,989 proteins remained in the final dataset. This suggests that the rate of data loss due to filtering and clustering is low, at around 3.9 %.

### Functional annotation

**Fig. 3:**
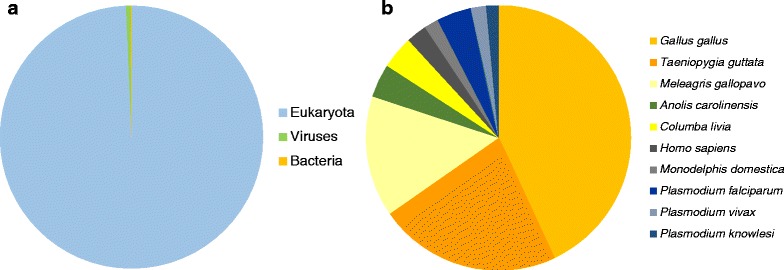
Taxonomic distribution of the top BLAST hits of transcripts. Hits are classified to the non-redundant database classified into: **a**) Eukaryotes, Bacteria and Viruses; **b**) the top ten species to which matches were recovered

96.1% of transcripts showed sequence homology to the NCBI non-redundant database. Of these, 99.3 % showed homology to Eukaryotes, 0.6 % to viruses and 0.1 % to bacteria (Fig. 3). The majority of top matches were to chicken, zebra finch and wild turkey (*Meleagris gallopavo*), accounting for 67.4 % of all hits (36.3 %, 18.7 % and 12.4 % respectively). The ten most commonly matched species also included an anole lizard (*Anolis carolinensis*), the rock dove (*Columba livia*), human (*Homo sapiens*), an opossum (*Monodelphis domestica*) and three malarial parasites of the genus *Plasmodium* (*P. falciparum*, *P. vivax* and *P. knowlesi*; see Fig. 3).

**Fig. 4:**
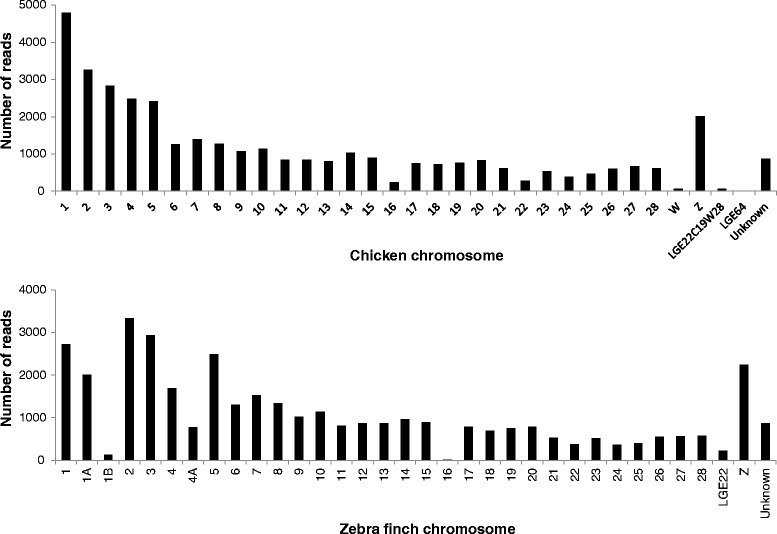
Chromosomal distribution of homologues to all transcripts with top BLAST hits to the chicken (*top panel*) and zebra finch (*bottom panel*). Designations starting with “LK” indicate linkage groups not assigned to chromosomes

**Fig. 5:**
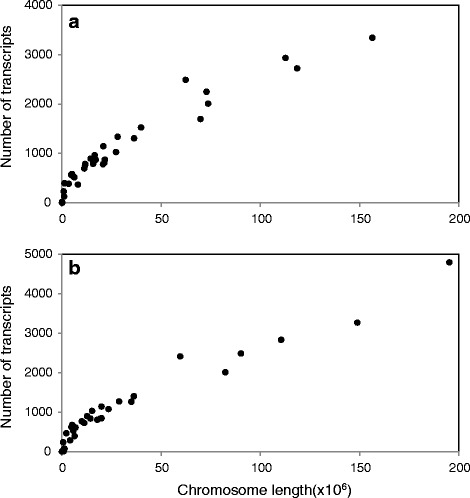
Relationship between the number of transcripts mapping to a chromosome and chromosome length. Mapped to in a) zebra finch (*r* = 0.96, *P* < 0.0001) and b) Chicken (*r* = 0.97, *P* < 0.0001)

We BLASTed the transcripts to chicken and zebra finch proteins with known genomic locations with a stringent e-value threshold of 10^-5^, revealing a broad genome-wide distribution of the genes (Fig. 4). As expected, relatively few mappings were obtained to the small W chromosome as well as to chromosome 16, a micro-chromosome that harbours the highly variable Major Histocompatibility Complex (MHC), which has been shown to have low synteny between chicken and other birds [44–47]. Overall, strong positive correlations were obtained between the number of reads mapping to a given chromosome and the length of that chromosome in both the chicken and zebra finch (*r* = 0.97, *P* < 0.0001 and *r* = 0.96, *P* < 0.0001 respectively, see Fig. 5). This implies that the amount of transcript sequencing effort for each chromosome was roughly in proportion to its length, and hence that the genomic distribution of the transcripts is fairly even. In total 38,535 transcripts (89.9 %) could be annotated based on similarity to annotated genes in either the chicken or zebra finch.

**Fig. 6:**
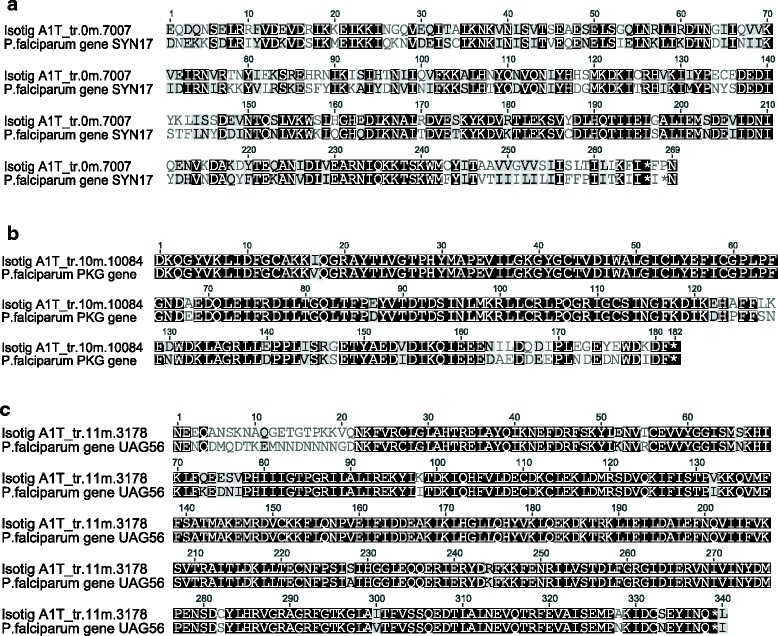
Alignments of translated protein sequences between three representative genes of *Plasmodium falciparum* and their homologous contigs from the *Buteo-Leucocytozoon* transcriptome. *SYN17*, *PKG* and *UAG56* have pairwise identities of 58 %, 87 % and 90 %, respectively. Alignment graphs were produces with Geneious 8.1 [63]

Given the high frequency of top matches to *Plasmodium* and the fact that individuals in our buzzard study population are sometimes heavily infected with *Leucocytozoon buteoni*s, a close relative of *Plasmodium*, we investigated further. Matches were found to a total of 12 *Plasmodium* species, accounting for 9.5 % of all top matches found (Table 1). Only a single transcript had its strongest match with *Leucocytozoon sp*. The greatest representation of transcripts matching to *Plasmodium* and/or *Leucocytozoon* was found in *organs* (1,905 transcripts, accounting for 18.1 % of transcripts in this pool). *Blood* also contained a high percentage of *Plasmodium*-like transcripts (681 transcripts, 11.7 %). In contrast, *Plasmodium*-like transcripts were nearly absent from feathers, representing only 0.1 % of all transcripts found in *dorsal* and *ventral feathers* combined. Of the 3,902 transcripts matched to *Plasmodium* or *Leucocytozoon*, 3,871 could be annotated with the NCBI RefSeq of *P. falciparum*, *P. knowlesi*, and/or *P. vivax*. Of these, 2,217 matched to known genes in *P.falciparum*, 2,205 in *P. knowlesi* and 2,190 in *P. vivax*. Examples of homology between *Leucocytozoon* transcripts and *P.falciparum* genes are presented in Fig. 6. The mean identity score of translated protein sequences between *P.falciparum* genes and *Leucocytozoon* was 65.8 %.

**Table 1:** Number and overall percentages of transcripts showing sequence homology to *Plasmodium* species

Species	Number of matched transcripts	Percentage of matched transcripts
*P. falciparum*	1463	3.550
*P. vivax*	646	1.568
*P. knowlesi*	512	1.243
*P. yoelii*	385	0.934
*P. cynomolgi*	347	0.842
*P. berghei*	288	0.699
*P. chabaudi*	215	0.522
*P. gallinaceum*	36	0.087
*P. reichenowi*	3	0.007
*P. ovale*	3	0.007
*P. malariae*	2	0.005
*P. vinckei*	1	0.002
Total	3901	9.467

### Molecular marker discovery

We mined transcripts with homology to vertebrate sequences separately from those that matched *Plasmodium*. In the former, we found 7,636 microsatellites distributed across 5,794 contigs, with a maximum of nine microsatellites per contig (Table 2). 5,216 (90 %) of these contigs were functionally annotated with respect to either the chicken or zebra finch. In transcripts with sequence homology to *Plasmodium spp*., 1,309 microsatellites were identified. These were distributed across 931 different contigs, each of which carried between one and five microsatellites (Table 2).

**Table 2:** Number of microsatellite markers found in transcripts with top BLAST matches to either vertebrates or blood parasites of the genus *Plasmodium*

Species group transcripts matched to	Repeat motif length	No. of repeats	% of all microsatellites
Vertebrates	2	2918	38.2
	3	4278	56.0
	4	249	3.3
	5	191	2.5
	Total	7636	
Plasmodium	2	728	55.6
	3	540	41.3
	4	31	2.4
	5	10	0.8
	Total	1309	

Searching the dataset for Single Nucleotide Polymorphisms (SNPs) revealed 96,966 SNPs residing in transcripts matched to vertebrates and 15,072 SNPs in transcripts matched to *Plasmodium*. After we filtered for a minimum Phred quality score of 15, there remained 64,907 and 10,067 SNPs respectively. Of all 36,722 transcripts matching to vertebrate sequences, 17,332 contained SNPs. The distribution of SNPs across contigs was fairly even, with just 303 transcripts containing >20 SNPs. On average, transcripts containing more than 20 SNPs were three times as long as those containing fewer SNPs.

### Differential expression patterns and melanin pathway characterisation

**Fig. 7:**
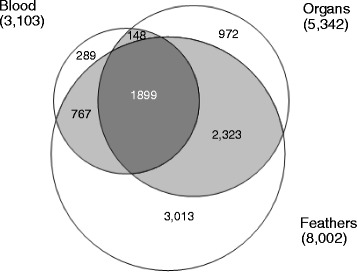
Venn diagram showing the differential representation of transcripts among the three pools containing samples from *blood*, *feathers* (*dorsal* and *ventral*) and *organs*, based on zebra finch annotations. Numbers in brackets indicate the total number of genes in a pool

To gain a broad overview of the representation of various transcripts in different tissues, we compared the number of zebra finch homologues assigned to the different pools (o*rgans*, *blood* or *feathers*, Table 3, Fig. 7). As the libraries had been normalised, precluding a quantitative comparison, we focused on tissue-specific patterns of transcript presence / absence (see Methods for details). The largest number of zebra finch homologues was found in *ventral feathers* (7,465), followed by *dorsal feathers* (6,208 genes), the *organs* pool containing lung, liver, skin, heart muscle, breast muscle and brain tissue (5,342), and finally *blood* (3,103). Combining the two *feather* types yielded a pool of 8,002 genes. This combined pool had the largest number of unique genes (3,013) followed by *ventral feathers* (1,041) and *organs* (972).

**Table 3:** Numbers of hits to known genes of transcripts obtained from different tissues. A transcript was assigned as being associated with melanogenesis if it showed sequence homology to one of 86 proteins from the melanin pathway of the zebra finch (KEGG database)

Pool	Overall	Unique to pool	Unique to pool [%]	Associated with melanogenesis	% of all genes associated with melanogenesis
*Blood*	3103	289	9.3	184	23.7
*Organs*	5342	972	18.2	420	54.0
*Ventral*	7465	1041	13.9	586	75.3
*Dorsal*	6208	318	5.1	497	63.9
*Feathers* (*Ventral* + *Dorsal*)	8002	3013	37.7	639	82.1
Total	9411	2620	27.8	778

To characterise genes involved in melanogenesis, we identified a total of 2,648 transcripts from all four pools showing homology to genes from the zebra finch melanin pathway. These transcripts were annotated by reference to the zebra finch RefSeq database, resulting in 778 hits to known genes (there was considerable redundancy in these data due to different transcripts having their best hits to different parts of the same gene). Of these, 586 (75.3 %) were from *ventral feathers* and 497 (63.9 %) were from *dorsal feathers*, while the combined *feather* pool accounted for 639 (82.1 %) hits. 53 of these were only found in *dorsal* but not in *ventral* feathers, whereas the opposite was true for 142 hits. *Organs* and *blood* contained 420 and 184 melanogenesis-related transcripts respectively (Table 3).

In order to identify candidate genes that could provide a link between plumage colouration and fitness, we identified melanogenesis-related transcripts that were differentially expressed in *dorsal* (dark) and *ventral* (light) feathers. As fitness-relevant genes are expected to be expressed not only in feathers, we then cross-referenced these transcripts with the *organs* pool to derive two subsets of transcripts from the melanogenesis pathway, one occurring in only *ventral* feathers and *organs*, and the other occurring in only *dorsal* feathers and *organs*. The resulting transcripts were then filtered for those with annotations relating to biological processes and categorised using AmiGO slimmer (www.geneontology.org; generic GO slim). Thirteen of 47 transcript GO categories exhibited large differences (i.e. >50 % difference in the relative number of genes represented) between the *dorsal* and *ventral feather* pools. These differences were attributable to 28 genes in the zebra finch annotation (Table 4). The greatest, and highly significant, difference was found for transcripts associated with immune system processes, which constituted only 1.8 % of genes represented in both *ventral feathers* and *organs* as compared to 12.3 % of genes represented in both *dorsal feathers* and *organs*.

**Table 4:** Transcripts associated with melanogenesis that occur in either *organs* and *ventral feathers* or *organs* and *dorsal feathers* (see Methods for details). A transcript was assigned as being associated with melanogenesis if it showed sequence homology to one of 86 proteins from the melanin pathway of the zebra finch (KEGG database)

Symbol	Name	GO slim categories	Characterized in	Best BLAST score against *Gallus gallus*
ADORA2A	Adenosine A2a Receptor	1,5,8	*Mus musculus*	
ARF1	ADP-Ribosylation Factor 1	12	*Mus musculus*	6E-96
BSG	Basigin	5	*Gallus gallus*	
CALM	Calmodulin	5	*Gallus gallus*	3E-76; 6E-82
FZD3	Frizzled 3	2,4,13	*Gallus gallus*	9.00E-86
FZD4	Frizzled 4	8,9,13	*Gallus gallus*	0.00E + 00
HRAS1	GTPase HRas	2,5,6,8,12	*Gallus gallus*	
LATS1	Large Tumor Suppressor Kinase 1	3,6	*Rattus norvegicus*	
LIMK1	LIM domain kinase 1	6,11,13	*Gallus gallus*	6.00E-174
LOC771574	(uncharacterised protein)	7	*-*	0.00E + 00
MAPKAPK2	Mitogen-activated protein kinase-activated protein kinase 2	1,12	*Rattus norvegicus*	0.00E + 00
NEK2	NIMA-Related Kinase 2	3,6	*Arabidopsis thaliana*	1.00E-106
OPN3	Opsin 3	9	*Mus musculus*	8.00E-154
PAK2	P21 Protein (Cdc42/Rac)-Activated Kinase 2	13	*Mus musculus*	0.00E + 00
PBRM1	Protein polybromo-1	3,8	*Gallus gallus*	
PLCL1	Phospholipase C-Like 1	7	*Mus musculus*	0.00E + 00
PLK2	Polo-Like Kinase 2	9	*Mus musculus*	9E-57; 2E-64
RAB11A	Ras-related protein Rab-11A	10,12	*Gallus gallus*	1.00E-117
RAB8A	RAB8A, Member RAS Oncogene Family	4,6,10,12,13	*Mus musculus*	3.00E-84
RAC1	Ras-Related C3 Botulinum Toxin Substrate 1	2,6,7,10,12,13	*Rattus norvegicus*	3.00E-111
RALA	Ras-Related Protein Ral-A	6,12	*Rattus norvegicus*	
SAR1A	SAR1 Homolog A	12	*Rattus norvegicus*	1.00E-115
Sox9	Sox9	1,2,3,4,6,8,11,13	*Gallus gallus*	5.00E-65
STK4	Serine/threonine-protein kinase 4	1,4,11,13	*Gallus gallus*	
SYK	Tyrosine-protein kinase SYK	1,2,5,7,8,12	*Gallus gallus*	1.00E-95
TTBK2	Tau Tubulin Kinase 2	4,6,13	*Mus musculus*	
VAV3	Vav 3 Guanine Nucleotide Exchange Factor	1,2,6,7,12	*Rattus norvegicus*	
WNT4	Protein Wnt-4		*Gallus gallus*	9.00E-57

1: Immune system process; 2: Cell motility; 3: Chromosome organisation; 4: Anatomical structure formation involved in morphogenesis; 5: Small molecule metabolic process; 6: Cellular component assembly; 7: Lipid metabolic process; 8: Biosynthetic process; 9: Neurological system process; 10: Membrane organisation; 11: Growth; 12: Vesicle-mediated transport; 13: Cell morphogenesis

### qPCR analysis

**Fig. 8:**
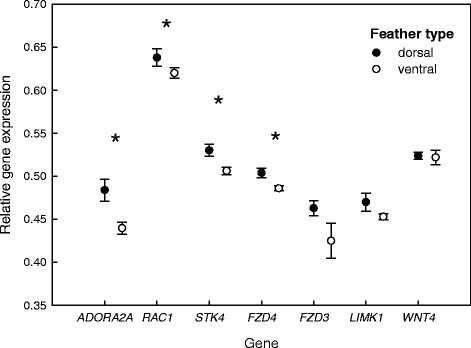
Results of the qPCR analysis showing the relative expression levels of seven melanogenesis-related genes in dorsal and ventral feathers. Normalised inverse Cq-scores (mean ± SE) are shown for seven candidate genes after normalisation relative to *Clock* (see Methods for details). These results are based on three common buzzard individuals for which both dorsal and ventral feather samples were available. Asterisks indicate significant differences between dorsal and ventral feathers for the respective gene

In order to explore these expression differences further, we conducted qPCR on 20 samples comprising 11 ventral and 9 dorsal feathers sampled from 7 dark, 2 intermediate and 8 light buzzards (see Additional file 1: Table S1). For three of the birds, we had RNA samples available from both dorsal and ventral feathers, allowing a direct comparison. We analysed seven of the genes identified by the differential expression analysis, together with *Clock* as a control that is unrelated to melanogenesis. After normalising the genes relative to *Clock*, we found that four genes (*ADORA2A*: *F*_1,8_ = 7.097, *P* = 0.008; *FZD4*: *F*_1,12_ = 4.638, *P* = 0.031; *RAC1*: *F*_1,8_ = 4.460, *P* = 0.035 and *STK4*: *F*_1,14_ = 4.294, *P* = 0.038) were significantly differentially expressed between ventral and dorsal feathers, two of which are immune genes (Fig. 8).

**Fig. 9:**
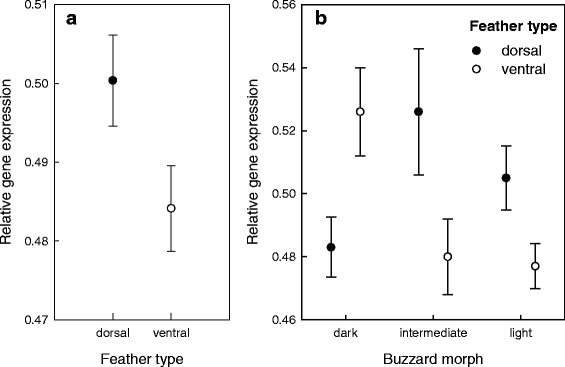
Results of the qPCR analysis showing the relative expression levels of seven candidate genes by feather type and morph. Normalised inverse Cq-scores are shown for seven candidate genes after normalisation relative to *Clock*. These results are based on the analysis of 20 samples within a GLM framework (see Methods for details). **a** mean ± SE expression levels of the seven genes in dorsal and ventral feathers; **b** mean ± SE expression levels of the seven genes in the dorsal and ventral feathers of the three colour morphs

To explore broad patterns, we constructed a general linear model of expression level, in which we fitted feather and morph as predictor variables together with their interaction. We found a significant effect of feather type (*F*_1,248_ = 5.702, *P* = 0.018), with dorsal feathers having higher expression levels than ventral feathers (Fig. 9a), together with a significant interaction between feather and morph (*F*_2,248_ = 4.302, *P* = 0.016). This is depicted in Fig. 9b, which shows that the scored genes have higher expression levels in dorsal versus ventral feathers in intermediate and light buzzards, but that this pattern is reversed in dark buzzards, where expression levels are higher in the ventral feathers.

## Discussion

### Assembly and functional annotation

We *de novo* assembled transcriptomes of the common buzzard and its blood parasite *L. buteonis*, identified species-specific molecular markers, and generated a list of candidate genes that could provide a link between plumage morph and fitness through impacts on various biochemical pathways, several of which are involved in immunity. We thereby provide a starting point for future molecular studies of the Accipitriformes, an ecologically distinctive and interesting group.

The newly assembled buzzard sequences obtained from a single sequencing run cover 67.3 % and 72.3 % of all protein sequences described in chicken and zebra finch respectively. The actual number of genes present in accipitriform species is unknown, but it is reasonable to assume based on comparison to these model avian species that a considerable fraction of common buzzard genes are represented in our dataset. Recently published data on peregrine and saker falcons [28] indicate that similar numbers of genes are also present in falconiforms. We therefore corroborate earlier studies suggesting that sampling from just one [48, 49] or a small number of different tissues [49–52] can provide reasonable coverage of the transcriptome. This is supported by the broad chromosomal distribution of genes matched to the buzzard transcripts in chicken and zebra finch and the strong positive correlations obtained between chromosome length and the number of matching transcripts in both species.

The distribution of zebra finch homologues across the three different tissue pools offers a number of insights that could be important for future studies of bird transcriptomes. First, *blood* contained a very small proportion of unique genes (only 9.3 % of genes detected in blood were unique to this pool), despite bird erythrocytes being nucleated and hence presumably transcriptionally active. As the blood samples yielded the lowest overall concentration of RNA, this could suggest either a technical artefact or limited transcriptional activity. The latter appears reasonably likely as most cells in the blood are erythrocytes. These produce mainly haemoglobins, one of the major transcripts we expected to reduce by normalising the RNA samples.

Second, we found a surprisingly large number of transcripts and genes that were unique to *feathers* in comparison to the *organs* pool, which comprised five different tissues (lung, liver, brain, muscle and skin). This may reflect the rapid growth rate of developing feathers, which in turn requires high transcriptional activity. In comparison, the internal organs will have been experiencing slower growth rates given the advanced age of the chick from which they were sampled. A similar, if slightly lower, number of expressed genes was found in growing feathers of Ruffs, *Philomachus pugnax* [51, 53].

An important caveat, however, to comparisons involving different pools is that the *blood*, *feathers* and *organs* pools were constructed from different sample sizes of individuals, ranging from one to 30. This could affect the diversity of transcripts, as one would expect to be able to capture more transcripts from a larger sample size of individuals due to inter-individual variation in gene expression. This could partly explain the observation that more zebra finch homologues were detected in *feathers* than in *organs*. However, there does not appear to be a clear relationship between the number of individuals in the pools and transcript diversity as the *blood* pool, which was based on the largest number of individuals, had the lowest diversity of transcripts.

### Parasite transcripts

In addition to generating a near-complete common buzzard transcriptome, we also obtained the most complete genomic resource to date for a *Leucocytozoon* species, a widespread group of avian malaria-like blood parasites. The closest relatives with well annotated genomes are three *Plasmodium* species, with 5,512, 5,506 and 5,161 annotated genes in *P. falciparum*, *P. vivax* and *P. knowlesi* respectively (NCBI database). Assuming that the number of genes is similar in *L. buteonis*, the 2,190–2,217 genes we found represent approximately 40–43 % of the full gene set. However, the recognition of species-specific genes will strongly depend on the available genomic and transcriptomic resources to BLAST against and how closely related they are to the target organism. Since *L.buteonis* belongs to a genus evolutionarily well separated from the reference *Plasmodium* genomes, the transcriptomic resource we provide probably contains more *Leucocytozoon*-specific genes than we can currently identify.

Most of the transcripts matching *Plasmodium* that were assigned to a single pool were found in the *organ* pool rather than in *blood* (73.1 % versus 26.1 %). One explanation for such a pattern is that fewer genes might be expressed during life cycle stages occurring in the host’s blood than during those inside the liver. This is unlikely, however, as over 60 % of *Plasmodium* genes are active during the intraerythrocytic stage [54]. Alternatively, the infection of the buzzard chick that we sampled may have been quite recent, with most of the parasites still developing and dividing inside the liver and only few merozoites having already been released into the blood stream. In other transcriptomic studies of birds, very few endoparasite transcripts have so far been found, presumably due to the fact that the main organs or tissues harbouring the parasites have not been sampled [55, 56].

Ours is the first study to our knowledge to have jointly characterised the transcriptomes of a vertebrate host and its parasite, in our case using only a single MiSeq lane (but see [64]). This has positive implications for the development of parasite genomic resources across a variety of taxa, and may also be beneficial for exploring host-parasite interactions. As most of the transcripts were derived from the organs pool, we do not yet know which of the organs contained the most transcripts, although it seems likely that this would be the liver. If so, future efforts to characterise malaria-like parasites should be able to achieve much greater sequence coverage by focusing on this organ. Finally, it is unclear if our success in obtaining parasite transcripts could be due to our having normalised the libraries. This would make good sense if the parasite transcripts are expressed at low levels relative to the host, because normalisation improves the extent to which rare transcripts are represented in the final sequencing libraries. Future work should make further refinements to our protocol in order to maximise the representation of both parasite and host transcripts.

By sequencing more than 40 % of all expected *Leucocytozoon* genes, we also offer the first transcriptomic basis for population genetic and phylogenetic studies of this parasite genus, whose relatedness to other hemosporidian parasites has been controversial [57]. Additionally, dual host and parasite transcriptomes should allow detailed studies of the genetics of host-parasite coevolution. Nonetheless, sequence similarity between the designated *Leucocytozoon* transcripts and the *Plasmodium* genome showed a wide variation with a mean of 66 % which is substantially lower than the 80–83 % similarity of mitochondrial sequences of *L.buteonis* to *Plasmodium* parasites [41]. This may hint toward different mutation rates between mitochondrial and nuclear genes [57, 58] but could also be the result of error-prone alignment of underrepresented parasite reads.

### Candidate genes for fitness differences

We attempted to identify genes potentially linking plumage melanisation to the fitness differences observed among the three colour morphs. For the transcriptome analysis, a direct comparison between individuals of different morphs was not possible due to constraints on the number of libraries we could produce. We therefore used the two different feather types as tentative proxies to gain a first impression of potentially important differences. While *dorsal* and *ventral* feathers differ not only in their melanisation but also in the body regions from which they were obtained, restricting comparisons to genes associated with the melanin pathway should be effective in focusing our analysis on genes relevant to colour differences.

For this analysis, we compared *ventral* (light) and *dorsal* (dark) feathers for the differential recovery of transcripts involved not only in melanogenesis in feathers, but also in other pathways that are expressed in other tissues. As the libraries had been normalised to maximise the overall representation of different transcripts, it would not have been appropriate to carry out a quantitative analysis of differential gene expression [59]. We therefore focused on tissue-specific patterns of transcript presence / absence. This approach is highly conservative in the sense that it should detect only the most extreme differences in expression, but we nevertheless recovered some interesting patterns. In particular, the greatest expression differences were observed for transcripts with GO annotation terms relating to immunity. Six immune-related transcripts were represented only in the darker *dorsal* feathers whereas one was found only in the lighter *ventral feathers*. This is consistent with the fact that dark buzzards tend to better suppress their *L. buteonis* infections than light ones [11], and provides the first genetic support for a putative link between melanisation and the immune response in vertebrates [19, 60].

Melanogenesis-related transcripts present in the *organs* pool as well as in either *ventral* or *dorsal feathers* are involved in a total of 56 different biological pathways. While it is too early to speculate on their role in generating fitness differences, we highlight four of these pathways and their components as potentially interesting candidates for further study. Each of these pathways is represented by several genes differentially expressed in *dorsal* and *ventral feathers*. Two of the pathways are involved in cell motility, including white blood cell migration during immune reactions (regulation of the actin cytoskeleton and focal adhesion). The central pathway connecting each of the three others is that for MAPK signalling, which is part of a conserved cascade involved in stress responses and regulation of gene expression. It connects to the WNT signalling pathway, which in turn regulates gene transcription and the cell cycle. Interestingly, both the MAPK and WNT signalling pathway also involve the cAMP response element binding protein (CREB), a transcription factor recently linked to differences in dispersal timing of the three buzzard morphs in our study population [20]. CREB is also involved in the regulation of neuropeptides (reviewed in [61, 62]) and therefore provides a potential link to behavioural differences between the morphs [21]. Consequently, although we can only speculate on the role of these pathways based on currently available data, the patterns that emerge are plausibly related to relevant biological processes, suggesting that our candidate gene set provides an interesting avenue for further study.

### qPCR analysis

To further explore patterns of differential expression in relation to both feather and morph, we conducted a qPCR analysis of seven genes selected from Table 4. We found significant differences in expression levels between dorsal and ventral feathers at four genes, including two immune genes (*ADORA2A* and *STK4*). qPCR analysis suggests that both of these genes are significantly upregulated in dorsal feathers, consistent with the fact that transcripts associated with immune system processes constituted only 1.8 % of genes represented in both *ventral feathers* and *organs* as compared to 12.3 % of genes represented in both *dorsal feathers* and *organs*. Furthermore, we found a significant interaction between feather type and morph. This interaction should be interpreted with caution due to the relatively small sample sizes involved. However, the selected genes appear to be expressed at a higher level in dorsal feathers in both intermediate and light buzzards, whereas the reverse appears to be the case for dark birds. Higher levels of expression in dorsal feathers might be expected as these feathers are on average darker than equivalent ventral feathers, particularly in the light and intermediate morphs (Fig. 1). However, in dark birds the breast feathers are comparatively darker.

## Conclusion

A single MiSeq run yielded enough sequence data to characterise a large proportion of the transcriptome of the common buzzard and its malaria-like *Leucocytozoon* parasite. Comparable to a recent study based on fur seals that died of natural causes [49], we also show that transcripts from a single dead animal represent 56.8 % of all the genes documented in all four libraries combined. This is a promising result for species of conservation concern in which non-destructive and/or non-invasive sampling is desirable. We not only identify several putative candidate genes for fitness differences relating to melanisation in the common buzzard, but we also provide a resource that should facilitate studies of other accipitriform species and the malaria-like *Leucocytozoon* parasites of birds in general.

### Ethics statement

All animal handling was performed with permission from the local authority Kreis Gütersloh, permit nr: 4.5.2-723-Bussard in accordance to German federal and state laws.

### Data accessibility

Raw sequence reads have been deposited at the European Nucleotide Archive (accession no. PRJEB5722) and can be found at http://www.ebi.ac.uk/ena/data/view/PRJEB5722.

## Additional file

Additional file 1: Table S1. Sample sizes used for qPCR according to the morph of the donor buzzard and the body region of sampling. For two light and one intermediate birds both ventral and dorsal feathers were available. (DOC 29 kb)
