# Supplementary material for: De novo assembly of the dual transcriptomes of a polymorphic raptor species and its malarial parasite
Source: BMC Genomics. 2015 Dec 9;16:1038. doi: 10.1186/s12864-015-2254-1 (PMC4673757; doi:10.1186/s12864-015-2254-1)
Supplement: Additional file 1: Table S1. — Sample sizes used for qPCR according to the morph of the donor buzzard and the body region of sampling. For two light and one intermediate birds both ventral and dorsal feathers were available. (DOC 29 kb) [file 12864_2015_2254_MOESM1_ESM.doc]

**Additional file 1 Table S1 Sample sizes used for qPCR according to the morph of the donor buzzard and the body region of sampling. For two light and one intermediate birds both ventral and dorsal feathers were available.**

|  | Origin of feather | | |
| --- | --- | --- | --- |
|  | Ventral | Dorsal | Total |
| Morph of donor |  |  |  |
| Dark | 1 | 6 | 7 |
| Intermediate | 2 | 1 | 2 |
| Light | 8 | 2 | 8 |
| Total | 11 | 9 |  |
